# Epidemiology and Genetic Diversity of Colistin Nonsusceptible Nosocomial* Acinetobacter baumannii* Strains from Russia for 2013-2014

**DOI:** 10.1155/2017/1839190

**Published:** 2017-10-17

**Authors:** Eugene A. Sheck, Mikhail V. Edelstein, Marina V. Sukhorukova, Natali V. Ivanchik, Elena Yu. Skleenova, Andrey V. Dekhnich, Ilya S. Azizov, Roman S. Kozlov

**Affiliations:** Institute of Antimicrobial Chemotherapy (IAC), Smolensk State Medical University (SSMU), Kirova St. 46a, Smolensk 214019, Russia

## Abstract

A high level of resistance to carbapenems in* Acinetobacter baumannii* strains severely limits therapeutic possibilities. Colistin is the last resort drug against such strains, although the cases of resistance to this drug have become more frequent. This article presents the epidemiological features and genetic diversity of colistin nonsusceptible* A. baumannii *strains collected as part of a national multicenter epidemiological study of the antibiotic resistance of pathogens of nosocomial infections (MARATHON), which was conducted in 2013-2014 in Russia. A total of 527* A. baumannii *isolates were collected, 10 (1.9%) of which were nonsusceptible to colistin. The majority of nonsusceptible* A. baumannii* isolates to colistin showed resistance to carbapenems and had the genes of the acquired OXA-40-like carbapenemases (*n* = 6). In one case, a combination of OXA-23-like + OXA-40-like (*n* = 1) genes was identified. One strain had the multidrug-resistant (MDR) phenotype, 6 isolates had extensively drug-resistant (XDR) phenotype, and 3 isolates had pandrug-resistant (PDR) phenotype. Among the colistin nonsusceptible* A. baumannii *isolates, 6 individual genotypes were identified, most of which belonged to successful international clones (CC92^OXF^/CC2^PAS^, *n* = 4; CC944^OXF^/ST78^PAS^, *n* = 4; CC109^OXF^/CC1^PAS^, *n* = 1).

## 1. Introduction

*Acinetobacter baumannii* is one of the most troublesome pathogens of nosocomial infections [1, 2]. This pathogen is characterized by intrinsic resistance to a number of drugs, as well as an outstanding ability to acquire the determinants of antibiotic resistance through horizontal gene transfer, which led to a high level of* A. baumannii* strains resistance to almost all available antibiotics [3, 4]. A high level of* in vitro *sensitivity is retained only for colistin [5], which makes it possible to use it against the extensively drug-resistant* A. baumannii* strains [6]. At the same time, the cases of resistance to colistin become more frequent [7–9], which determines the necessity to monitor resistance to colistin among nosocomial* A. baumannii* strains. In addition, the spread of the multidrug-resistant (MDR) phenotype is mainly related to the spread of international high-risk clones and the horizontal transfer of antibiotic resistance genes [10, 11], which provoke the interest in studying the molecular epidemiology of colistin nonsusceptible strains. The purpose of the research is to study the epidemiology and genetic diversity of colistin nonsusceptible* A. baumannii* strains, isolated from clinical samples of hospitalized patients in different regions of Russia in 2013-14.

## 2. Materials and Methods

Clinical strains of microorganisms were collected as part of a multicenter epidemiological surveillance study of the antibiotic resistance of nosocomial pathogens (MARATHON) [12] in 21 cities of Russia from January 2013 to December 2014. 527 nosocomial isolates of* A. baumannii* were collected. In this study, only colistin nonsusceptible strains (MIC > 2 mg/L) were analyzed.

Species identification was performed by the matrix-assisted laser desorption/ionization time-of-flight mass spectrometry (MALDI-ToF MS) using Microflex LT mass spectrometer and MALDI Biotyper Compass software v. 4.1.70 (Bruker Daltonics, Germany). The value of “Score” ≥ 2.0 was accepted as a measure for the reliable identification.

Minimal inhibitory concentrations of antimicrobials have been determined by broth microdilution method with Mueller Hinton broth (Oxoid, United Kingdom) in accordance with ISO 20776-1:2006 [13]. Interpretation of MIC in clinical susceptibility categories of* A. baumannii *isolates to antimicrobial agents was performed according to European Committee on Antimicrobial Susceptibility Testing (EUCAST) Breakpoint tables v.7.1 [14] (for aminoglycosides, carbapenems, ciprofloxacin, trimethoprim/sulfamethoxazole, and colistin) and also Clinical and Laboratory Standards Institute CLSI M100-S27 [15] (for penicillins with *β*-lactamase inhibitors and extended-spectrum cephalosporins). Isolates with tigecycline MIC of >1 mg/L were considered insusceptible to this drug [16].* Escherichia coli* ATCC®25922,* E. coli* ATCC®35218, and* Pseudomonas aeruginosa* ATCC®27853 were used as control strains.

DNA extraction was performed by express method by using InstaGene™ matrix (Bio-Rad, USA). Samples of extracted DNA were stored at −20°С before testing.

Detection of genes encoding the most common carbapenemases of class D (OXA-23, OXA-24/40, and OXA-58) and metal *β*-lactamases (VIM, IMP, and NDM) was carried out by real-time PCR using commercial kits “AmpliSens® MDR Acinetobacter-OXA-FL” and “AmpliSens® MDR MBL-FL” (InterLabService, Russia) and DTPrime 5X1 system (DNA Technology, Russia).* A. baumannii, A. pittii*, and* P. aeruginosa *strains from proper collection, producing known carbapenemases of the listed groups, were used as positive controls.

Genotyping was performed by single-nucleotide polymorphism (SNP) typing method [17]. Briefly, this method is based on analysis of 21 informative SNPs in 10 chromosomal loci* (gltA*,* recA*,* cpn60*,* gyrB*,* gdhB*,* rpoD*,* fusA*,* pyrG*,* rplB*, and* rpoB)* and is used at the University of Oxford and the Pasteur Institute multilocus sequence typing (MLST) schemes.* A. baumannii *strains of known sequence types from proper collection were used as positive controls. The comparison of received SNP profiles with MLST data was done using a web resource: http://snptab.antibiotic.ru [18]. Cluster analysis of SNP profiles was performed using PHYLOViZ 2.0 software (http://www.phyloviz.net/).

## 3. Results and Discussion

Ten of 527 (1.9%) nosocomial colistin nonsusceptible* A. baumannii* isolates were isolated from 7 hospitals in 7 cities of Russia in 2013-2014. The results of antimicrobial susceptibility testing for these isolates are presented in Table 1. The MIC of colistin 4 mg/L was detected in 1 isolate, while the remaining isolates (*n* = 9) had high levels of resistance (MIC range: 32–256 mg/L).

The majority of colistin nonsusceptible* A. baumannii* isolates were also resistant to carbapenems and were the carriers of genes of acquired OXA type carbapenemases, mainly OXA-40-like (*n* = 6). One isolate was shown to possess simultaneously genes encoding two different carbapenem hydrolyzing oxacillinases: OXA-23-like and OXA-40-like. Expectedly high resistance was detected to all other *β*-lactams. At the same time, associated resistance to drugs of other groups with high MIC values is observed (range of gentamicin MIC: 32–256 mg/L; amikacin: 16–256 mg/L; ciprofloxacin: 32–128 mg/L). Only one tobramycin susceptible isolate was found (MIC = 1 mg/L), while the MIC for the remaining isolates was in the range 16–256 mg/L. Two isolates were susceptible to combination of trimethoprim-sulfamethoxazole (MIC range: 1-2 mg/L), while for the remaining isolates the MIC was in the range 4–128 mg/L. Six isolates had tigecycline MICs of 2 to 8 mg/L and thus were considered as resistant.

In accordance with international criteria [19], 1 isolate was defined as multidrug-resistant (MDR), 6 isolates were defined as extensively drug-resistant (XDR), and 3 isolates were defined as pandrug-resistant (PDR).

Most infections associated with colistin nonsusceptible* A. baumannii* isolates were detected as single cases in different cities of Russia. An exception is Smolensk, from which 4 isolates were isolated in one hospital. In this connection, it seemed interesting to evaluate the population structure of studied strains.

**Figure 1:**
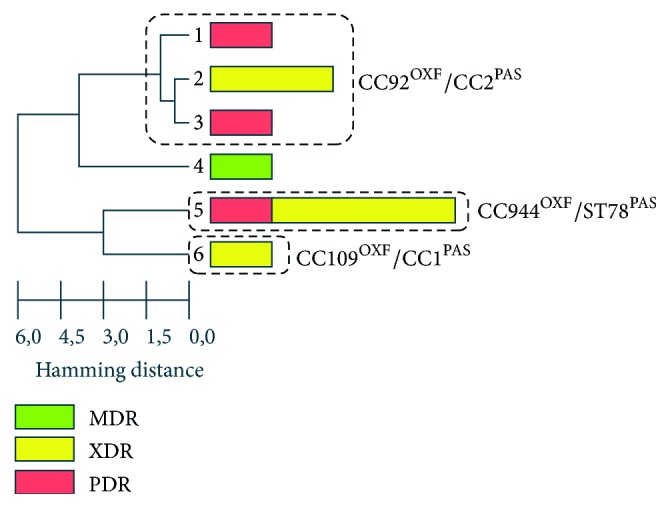
UPGMA clustering of SNP profiles of colistin nonsusceptible* A. baumannii* strains. The length of each rectangle depends on the number of isolates. International epidemic clones are marked with dashed lines. Color detailed the resistance categories (MDR, XDR, and PDR) of isolates within each genotype.

Colistin nonsusceptible* A. baumannii* isolates belonged to 6 genotypes (Figure 1). Three genotypes differed from each other in no more than 2 positions and were united in a single genetic cluster as related genotypes. This cluster included 4 isolates from 4 cities and corresponded to the international clone CC92^OXF^/CC2^PAS^ (according to the MLST schemes of the University of Oxford and the Pasteur Institute, resp.). Another genotype included 4 isolates from 3 cities and corresponded to CC944^OXF^/ST78^PAS^. The strains of this genetic lineage are widespread in the territory of Russia and Belarus [12] and, according to the literature, were found in Italy, the US, and Germany [20–23]. This allows considering this genotype as a new international clone. Two isolates belonged to individual genotypes: one of them corresponded to the international clone CC109^OXF^/CC1^PAS^ and the other to the clonal complex CC490^OXF^/CC25^PAS^. All isolates with XDR and PDR phenotypes belonged to the international epidemic clones CC92^OXF^/CC2^PAS^, CC944^OXF^/ST78^PAS^, and CC109^OXF^/CC1^PAS^.

## 4. Conclusion

Thus, colistin nonsusceptible* A. baumannii* strains described in this study relate to different genetic lineages and mainly belong to distinct international high-risk clones. The accumulation of molecular typing data is an important element in understanding the roots and epidemiology of colistin resistance and in predicting its further spread.

## Figures and Tables

**Table 1:** Characteristic of nosocomial colistin nonsusceptible *A. baumannii* strains.

Isolate	Source^*∗*^	City	Year	Clonal group	Carbapenemase	MIC (mg/L)/clinical category															Category
						Ampicillin/sulbactam	Piperacillin/tazobactam	Cefotaxime	Ceftazidime	Cefepime	Imipenem	Doripenem	Meropenem	Gentamicin	Tobramycin	Amikacin	Ciprofloxacin	Tigecycline	Colistin	Trimethoprim/sulfamethoxazole	
MAR13-1673	UT	Smolensk	2013	CC944^OXF^/ST78^PAS^	OXA-40-like	16	8	16	4	8	32	4	64	256	64	128	128	2	256	16	XDR
						I	S	I	S	S	R	R	R	R	R	R	R	R	R	R	
MAR13-1674	RT	Smolensk	2013	CC944^OXF^/ST78^PAS^	OXA-40-like	128	128	256	32	256	32	64	128	256	256	512	128	8	256	32	PDR
						R	R	R	R	R	R	R	R	R	R	R	R	R	R	R	
MAR13-1680	SST	Smolensk	2013	CC109^OXF^/*СС*1^PAS^	OXA-40-like	16	256	256	256	256	0.5	4	8	256	256	512	128	2	256	32	XDR
						I	R	R	R	R	S	R	I	R	R	R	R	R	R	R	
MAR13-1127	RT	Murmansk	2013	CC92^OXF^/*СС*2^PAS^	OXA-40-like	64	256	256	64	32	64	128	128	32	32	16	128	8	32	256	PDR
						R	R	R	R	R	R	R	R	R	R	I	R	R	R	R	
MAR13-1452	SST	Omsk	2013	CC92^OXF^/*СС*2^PAS^	OXA-23-like, OXA-40-like	256	256	256	256	256	32	128	64	256	256	512	128	4	256	256	PDR
						R	R	R	R	R	R	R	R	R	R	R	R	R	R	R	
MAR14-236	UT	Smolensk	2014	CC92^OXF^/*СС*2^PAS^	Negative	64	256	256	256	256	2	4	8	256	256	512	32	1	128	128	XDR
						R	R	R	R	R	S	R	I	R	R	R	R	S	R	R	
MAR14-1518	B	Tomsk	2014	CC92^OXF^/*СС*2^PAS^	Negative	64	256	256	256	256	2	1	1	256	1	256	64	4	128	2	XDR
						R	R	R	R	R	S	S	S	R	S	R	R	R	R	S	
MAR14-1542	SST	Tolyatti	2014	CC490^OXF^/CC25^PAS^	Negative	8	128	256	256	32	1	1	1	256	16	64	64	1	128	4	MDR
						S	R	R	R	R	S	S	S	R	R	R	R	S	R	I	
MAR14-2675	IA	Izhevsk	2014	CC944^OXF^/ST78^PAS^	OXA-40-like	256	256	256	256	256	128	64	128	256	256	512	128	0.5	128	128	XDR
						R	R	R	R	R	R	R	R	R	R	R	R	S	R	R	
MAR14-3743	RT	Penza	2014	CC944^OXF^/ST78^PAS^	OXA-40-like	32	256	256	128	128	32	32	64	256	256	512	128	0.5	4	1	XDR
						R	R	R	R	R	R	R	R	R	R	R	R	S	R	S	

^*∗*^UT: urinary tract; RT: respiratory tract; SST: skin and soft tissue; B: blood; IA: intra-abdominal.
